# Co-infection of Uropathogenic *Escherichia coli* among COVID-19 Patients Admitted to a Tertiary Care Centre: A Descriptive Cross-sectional Study

**DOI:** 10.31729/jnma.7376

**Published:** 2022-03-31

**Authors:** Ajaya Basnet, Arun Bahadur Chand, Lok Bahadur Shrestha, Nayanum Pokhrel, Lochan Karki, Sailendra Kumar Duwal Shrestha, Basanta Tamang, Mahendra Raj Shrestha, Maina Dulal, Junu Richhinbung Rai

**Affiliations:** 1 Department of Medical Microbiology, Shi-Gan International College of Science and Technology, Tribhuvan University, Kathmandu, Nepal; 2 Department of Clinical Laboratory, KIST Medical College and Teaching Hospital, Lalitpur, Nepal; 3 School of Medical Sciences and the Kirby Institute, University of New South Wales, Sydney, Australia; 4 Nepal Health Research Council, Kathmandu, Nepal; 5 Department of Medicine, National Academy of Medical Sciences, Bir Hospital, Kathmandu, Nepal; 6 Department of Orthopaedic and Trauma Services, Nepal Armed Police Force Hospital, Kathmandu, Bagmati, Nepal; 7 Department of Clinical Laboratory, Nepal Armed Police Force Hospital, Kathmandu, Nepal; 8 Department of Clinical Microbiology, Tribhuvan University Hospital, Kathmandu, Bagmati, Nepal

**Keywords:** *antimicrobial drug resistance*, *co-infection*, *COVID-19*, *Escherichia coli*, *procalcitonin*

## Abstract

**Introduction:**

Simultaneous infection of antibiotic-resistant uropathogens in patients with COVID-19 has necessitated the revision of the prescription of broad-spectrum antibiotics on the grounds of evidence-based studies and antimicrobial stewardship principles. The objective of this study was to find out the prevalence of uropathogenic *Escherichia coli* co-infection among hospital-admitted COVID-19 patients of a tertiary care centre.

**Methods:**

This descriptive cross-sectional study was conducted in urinary tract infection suspected COVID-19 patients admitted to a tertiary care hospital, from 25^th^ June to 24^th^ December 2021 after ethical clearance from the Institutional Review Committee with registration number 207707860. Convenience sampling was used. Serum procalcitonin levels were also measured. Data analysis was performed using the Statistical Package for the Social Sciences software version 17.0. Point estimate at 95% Confidence Interval was calculated along with frequency and proportion for binary data, and mean and standard deviation for continuous data.

**Results:**

Among the 49 hospital-admitted COVID-19 patients, 3 (6.12%) (0.59-12.83 at 95% Confidence Interval) were co-infected with uropathogenic *Escherichia coli.* Absolute non-susceptibility of *Escherichia coli* to antibiotics such as ceftriaxone, cotrimoxazole, nalidixic acid, gentamicin, and ampicillin was observed. All isolates were multidrug-resistant. All co-infected patients were female and had a median age of 35 years. Mean±SD value for procalcitonin in patients with co-infection (6.13+7.88 ng/ml) was six times higher than for the patients without co-infection (0.95+1.11 ng/ml).

**Conclusions:**

*Escherichia coli* co-infection in hospitalised COVID-19 patients was less frequent as compared to published literature. The serum procalcitonin value in patients with co-infection was substantially higher than that of patients without co-infection.

## INTRODUCTION

Bacterial co-infections have been a notable concern Severe Acute Respiratory Syndrome Coronavirus-2 in viral respiratory tract infections, as was evident (SARS-CoV-2), has officially been declared a worldwide in influenza, where bacterial infections account for almost 20-30% of the co-infections associated with severe influenza.^1^

COVID-19, caused by a novel coronavirus, namely, Severe Acute Respiratory Syndrome Coronavirus-2 (SARS-CoV-2), has officially been declared a worldwide pandemic by the World Health Organization in March 2020.^2^ SARS-CoV-2-induced lymphocytes damage of the host may lead to the impairment of respiratory and immune system, which could result in microbial coinfections among patients with COVID-19.^3,4^ However, there is limited literature, which focuses on uropathogenic co-infections, their patterns of antimicrobial resistance, and supporting the use procalcitonin for initiation of antibiotic treatments before the confirmation of coinfection in patients infected with SARS-CoV-2.^5,6^

Therefore, this study aimed to find the prevalence of uropathogenic *Escherichia coli* among COVID-19 patients admitted to a tertiary care centre.

## METHODS

A descriptive cross-sectional study was conducted among patients with COVID-19, who were admitted to KIST Medical College and Teaching Hospital (KISTMCTH), Gwarko, Lalitpur, Nepal, between June 25 and December 24, 2021. The study was approved by the Institutional Review Committee (Reference number: 207707860) of the KISTMCTH. Reverse Transcriptase-Polymerase Chain Reaction (RT-PCR) confirmed cases with the diagnosis of SARS-CoV-2 were admitted to the COVID ward of KISTMCTH and they were included in the study. The COVID-19 patients with inadequate documentation were excluded from this study. Convenience sampling was done.

The sample size was calculated using the following formula.

n = (Z^2^ × p × q) / e^2^

  = (1.96^2^ × 0.07 × 0.93) / 0.08^2^

  = 40

Where,

n = minimum required sample size
Z = 1.96 at 95% of Confidence Interval (CI)
p = prevalence of *E. coli* among COVID-19 patients taken from previous study, 7%^7^
q = 1-p
e = margin of error, 8%

The calculated sample size was 40. After adding a 10% non-response rate, the sample size was 44. However, a sample size of 49 COVID-19 patients meeting the selection criteria was taken.

Bacterial infections were defined according to clinical symptoms, increased white blood cell (WBC) count, procalcitonin or C-reactive protein (CRP) levels, and positive results of bacterial culture.^8^

The relevant demographic data and detailed medical history of the COVID-19 patients were collected from the hospital record section. Data collection of the age, sex, and serum procalcitonin level were done, upon clinical suspicion of urinary tract infection, midstream urine (MSU) samples were obtained from the patients who were admitted with the diagnosis of COVID-19. From all MSU samples, 1 y^l of urine was inoculated on Cysteine Lactose Electrolyte Deficient agar using the semi-quantitative method and incubated aerobically for 24 hours at 37°C. Quantitative enumeration of urine sample based upon the colony-forming unit (CFU) was performed, and culture growth was reported as insignificant growth for 104 CFU/ml organism, doubtful significance for 104-105 CFU/ml organisms (suggest repeat specimen), and significant bacteriuria for more than 105 CFU/ml organisms and was considered as culture positive. The culture-positive colonies were further identified for *E. coli* by macroscopic observation followed by identification with the standard microbiological procedures, including Gram's staining and biochemical tests. All urine samples were processed as infectious in Biosafety cabinet Class IIA/IIB, using standard microbiological procedures.

Antibacterial susceptibility test of the identified isolates was performed by Kirby Bauer's disc diffusion method on Mueller Hinton agar following the latest 30^th^ guideline of Clinical Laboratory Standards Institute (CLSI) 2020.^9^ For analysis of serum procalcitonin level, 2 ml of venous blood samples were drawn from the admitted COVID-19 patients. The serum was separated by centrifugation at 4000 rpm for five minutes. Serum procalcitonin was determined by an immunofluorescence assay (Fine Care Fluorescence Analyzer, China) in the laboratory of biochemistry.^10^

Data analysis was performed using the Statistical Package for the Social Sciences software version 17.0. Point estimate at 95% Confidence Interval was calculated along with frequency and proportion for binary data, and mean and standard deviation for continuous data.

## RESULTS

A total of 49 COVID-19 patients, who were clinically suspected of urinary tract infection, underwent a subsequent panel of microbiological investigations. Among 49 MSU samples received from each of the patients, 3 (6.12%) (0.59-12.83 at 95% Confidence Interval) of them were co-infected with uropathogenic E *coli* (Table 1).

**Table 1:** Microbiological culture isolated bacteria (n= 49).

Outcome	n (%)
Sterile	46 (93.88)
*E. coli*	3 (6.12)

Antibiotic sensitivity testing for *E. coli* showed a variable degree of resistance. Absolute resistance amongst *E. coli* was observed for commonly used antibiotics such as ceftriaxone, cefepime, ceftazidime, nalidixic acid, norfloxacin, gentamicin, ampicillin, and cotrimoxazole. However, *E. coli* isolates also showed absolute sensitivity towards polymyxin B, tigecycline, imipenem, and amoxicillin-clavulanic acid combination. All 3 (100%) *E. coli* isolates were multidrug-resistant (Table 2).

**Table 2:** Antibiotic resistance among uropathogenic E. coli (n= 3).

Class	Antibiotics	Sensitive n (%)	Intermediate n (%)	Resistant n (%)
	Ceftriaxone	-	-	3 (100)
Cephalosporins	Cefepime	-	-	3 (100)
	Ceftazidime	-	-	3 (100)
ß-lactams + ß-lactamase inhibitor	Amoxicillin + Clavulanic acid	3 (100)	-	-
	Cefotaxime + Clavulanic acid	1 (33.33)	1 (33.33)	1 (33.33)
	Piperacillin + Tazobactam	2 (66.66)	1 (33.33)	-
	Nalidixic Acid	-	-	3 (100)
Quinolone and its	Ciprofloxacin derivatives	-	1 (33.33)	2 (66.66)
	Ofloxacin	1 (33.33)	-	2 (66.66)
	Norfloxacin	-	-	3 (100)
Aminoglycosides	Gentamicin	-	-	3 (100)
	Amikacin	1 (33.33)	1 (33.33)	1 (33.33)
Polymyxins	Colistin	2 (66.66)	1 (33.33)	-
	Polymyxin B	3 (100)	-	-
	Ampicillin	-	-	3 (100)
	Imipenem	3 (100)	-	-
Others	Tigecycline	3 (100)	-	-
	Nitrofurantoin	2 (66.66)	1 (33.33)	-
	Cotrimoxazole	-	-	3 (100)

The median (IQR) age of the co-infected patients was 35 years. All 3 (6.12%) co-infected patients were female and belonged to the reproductively active age group of 30-40 years (Table 3).

**Table 3:** Socio-demographic and procalcitonin details of co-infected patients.

Patient number	Age (years)	Sex	Serum procalcitonin level (ng/ml)
Patient 1	35	Female	1.16
Patient 2	35	Female	14.08
Patient 3	36	Female	15.42

The mean±SD value for serum procalcitonin levels in patients with co-infection (6.13±7.88 ng/ml) was significantly higher than that for serum procalcitonin levels for patients without bacterial co-infection (0.95±1.11 ng/ml).

## DISCUSSION

Currently, COVID-19 remains a novel public health concern, and the future of this pandemic remains largely unknown. Globally, more than two hundred and ninety-six million cases of COVID-19 with more than five million deaths have been reported as of January 7, 2021,^11^ and a total of 8,30,480 confirmed cases with 11,602 deaths have been reported in Nepal as of January 7, 2021, the time period when this study was conducted.^12^ Though bacterial co-infections in previous influenza pandemics have been a major cause of mortality,^12^ the literature concerning microbiology of bacterial co-infection and its increased risk in COVID-19 patients remains an underexplored area.^13^

In this study, bacterial co-infections with uropathogenic E *coii* occurred in 6.12% of microbiologically analysed COVID-19 patients, which is in line with the findings of anothe study which had observed incidence rates varying from one to 10% from two different studies.^14^ Similarly, a study reported a 7.0% bacterial co-infection rate by uropathogenic *E coli* in COVID-19 patients, which is also comparable with our findings.^7^ However, the rate of co-infection with uropathogenic E *coii* in patients with COVID-19 was substantially lower in our study than previously observed (33.5%) from another study.^15^ While some studies have reported secondary infection rates of 5.1%^16^ and 9.8%,^17^ a multicentre study from Wuhan observed secondary infections as high as in 50% of COVID-19 patients,^18^ which is in contrast with our findings. Such variations might be due to analyses of bacterial co-infections due to multiple species isolated from several clinical samples.

Gram-negative bacteria has been frequently associated with healthcare-associated infections (30%), ventilator-associated pneumonia (47%), and urinary tract infections (45%) according to some studies.^19^ These bacteria are highly efficient to resist antibiotics, either by up-regulation or by the acquisition of genes that code for antimicrobial resistance mechanisms.^20^ The present study showed *E. coli* isolates being susceptible (100%) to higher antibiotics such as polymyxin B, imipenem, and tigecycline whereas, on the other hand, they showed 100% resistance to cephalosporins (ceftriaxone, cefepime, ceftazidime), norfloxacin, gentamicin, ampicillin, and cotrimoxazole. Concerning a high incidence of antibiotic resistance in *E. coli*, a study has revealed the occurrence of extended-spectrum S-lactamase (ESBL)-producing phenomenon among such pathogens.^20^ Inappropriate antibiotic prescription is one of the major factors associated with an increase in antimicrobial resistance.

The median age of the co-infected COVID-19 patients in this study was 35 years, which was discordant to the observations of other studies (≥50 years)^21^ and (53.1 years)^22^ who had simply discussed the median age of COVID-19 patients, unlike to our study that focuses on co-infection. Some of the reasons put forward for SARS-CoV-2 infection in the elderly is due to gradual weakening of the immune system, age-related modification in the respiratory system (structural changes, gas exchange abnormalities, and reduced respiratory muscle strength), and increased prevalence of comorbidities such as hypertension, diabetes, chronic obstructive pulmonary diseases, and others.^23^ The increased percentage of urinary tract infection in females as observed in this study could be attributable to several clinical factors including anatomical differences such as squatness of females' urethra resulting in easy contamination with faecal flora, pregnancy, hormonal effects, and behaviour patterns.^24^

In our study, the mean±SD value for serum procalcitonin in patients with co-infection was 6.13 ±7.88 ng/ml, which is inconsistent with the findings of another study,^20^ (1.62 ng/ml) determined from critically ill COVID-19 patients. In our study, we observed a substantial elevation in serum procalcitonin value among patients with bacterial co-infection (6.13±7.88 ng/ml) as compared to the patients without bacterial co-infection (0.95±1.11 ng/ml). The mean value of serum procalcitonin levels in patients with co-infection was six times higher than the patients without coinfection. Similarly, the rate of nosocomial bacterial infection in COVID-19 patients with procalcitonin value >0.1 ng/ml was 4.92 times more than the patients with procalcitonin value ≤0.1 ng/mL in a study.^22^ Such evidence coherently highlights the usefulness of elevated serum procalcitonin levels as a useful adjunct to guide the initiation and/or cessation of antibiotic therapy in infected patients.^1^ Additionally, a serum procalcitonin value of >0.5 ng/ml in patients without bacterial co-infection could be due to cross-reactions with some components of serum from individual to antibodies; and non-specific adhesion of some components in human blood that has similar epitopes to capture and detect antibodies.^25^ Therefore, though procalcitonin values serve as a crucial biomarker in enabling specific differentiation between a bacterial infection and other causes of inflammatory reactions, the results of this rapid test should be carefully interpreted and evaluated with the full knowledge of the patient's clinical and laboratory data available.

Our findings were based on a limited number of observational studies; therefore, further well-designed studies with larger sample sizes are necessary to be conducted. Additionally, findings on antibiotic use should be interpreted with caution as there may be selection bias.

## CONCLUSIONS

The co-infection of uropathogenic *E. coli* in COVID-19 patients admitted to the hospital was found to be lower than published literature. The serum procalcitonin value in patients with co-infection was higher than in patients without co-infection, which was similar to other studies.
